# Who’s Using PDAs? Estimates of PDA Use by Health Care Providers: A Systematic Review of Surveys

**DOI:** 10.2196/jmir.8.2.e7

**Published:** 2006-05-12

**Authors:** Chantelle Garritty, Khaled El Emam

**Affiliations:** ^3^Department of PediatricsFaculty of MedicineUniversity of OttawaOttawaONCanada; ^2^Department of Public Health ScienceFaculty of MedicineUniversity of TorontoTorontoONCanada; ^1^Chalmers Research GroupChildren’s Hospital of Eastern Ontario Research Institute (CHEO RI)OttawaONCanada

**Keywords:** Personal digital assistant, systematic review, survey, health care, health technology adoption

## Abstract

**Background:**

Personal digital assistants (PDAs) find many uses in health care. Knowing rates of collective PDA use among health care providers is an important guiding step to further understanding those health care contexts that are most suited to PDA use and whether PDAs provide improved health outcomes.

**Objectives:**

The objectives of this study were to estimate current and future PDA use among health care providers and to discuss possible implications of that use on choice of technology in clinical practice and research.

**Methods:**

This study was a systematic review of PDA usage surveys. Surveys were identified as part of an ongoing systematic review on the use of handheld devices. Reports from eight databases covering both biomedical sciences and engineering (1993-2006) were screened against distinct eligibility criteria. Data from included surveys were extracted and verified in a standardized way and were assessed descriptively.

**Results:**

We identified 23 relevant surveys, 15 of which were derived from peer-reviewed journals. This cohort of surveys was published between 2000 and 2005. Overall, since 1999, there is clear evidence of an increasing trend in PDA use. The current overall adoption rate for individual professional use ranges between 45% and 85%, indicating high but somewhat variable adoption, primarily among physicians.

**Conclusions:**

Younger physicians and residents and those working in large and hospital-based practices are more likely to use a PDA. The adoption rate is now at its highest rate of increase according to a commonly accepted diffusion of innovations model. A common problem with the evaluation of information technology is that use frequently precedes research. This is the case here, in which PDA adoption rates are already high and projections are for rapid growth in the short term. In general, it appears that professional PDA use in health care settings involves more administrative and organizational tasks than those related to patient care, perhaps signaling where the growth in adoption is most likely to occur. We conclude that physicians are likely accustomed to using a PDA, and, therefore, technology expertise will probably not be a barrier to implementing PDA applications. However, there is an urgent need to evaluate the effectiveness and efficiency of specific tasks using handheld technology to inform those developing and those using PDA applications.

## Introduction

A handheld computing device, also commonly known as a personal digital assistant (PDA), is a mobile computer about the size of the palm of the hand. More modern devices can access external networks or the Internet through a wireless connection. Since 1993, when Apple launched the first PDA (Newton MessagePad), use of PDAs has increased worldwide, with global PDA sales projected to surpass 17 million in 2008. This represents a compounded annual growth rate of 17.8% between 2002 and 2008 [1].

Health care has not been immune to this technological advance in handheld computing. In fact, PDAs find many applications in health care. Family physicians and specialists have been using PDAs for general medical reference, such as drug interactions, pharmacopeias, and cardiac risk [2-4]. Other important applications of PDAs are those involving data collection and management, as in patient tracking, electronic Case Report Forms in clinical trials, patient diaries, and infection surveillance [4-9]. However, the suitability of PDAs across all health care contexts and whether they benefit health outcomes remain open questions.

Many of us would agree that it is necessary to evaluate a technology before its adoption to allow health care providers to make informed decisions. However, given that technology is a moving target, a common problem with evaluation is that practice frequently precedes research. By the time researchers have obtained funding, completed a study, and published it, the technology is either in widespread use or has been abandoned [10]. As well, the appropriate type of evaluation is not independent of the stage of adoption of the technology. For example, if 90% of the target users have already adopted a technology, then studies evaluating its general utility will no longer inform the adoption decision. In this case, research should focus on optimization of the technology in use. This is a familiar scenario in information technology research, and it underscores the importance of understanding the rates of adoption in helping direct approaches to research [10].

In a general overview article, Fischer et al (2003) summarized the current literature covering the use of handheld devices in medicine, primarily related to PDA functionality [4]. While implementation issues were discussed, rates of adoption were not addressed. Further, a recent review of PDA use in health care by Baumgart (2005) examined operating systems, basic functionality, security and safety, and limitations of PDA use [11]. It is a thorough overview of studies published since 2000 that addresses applications of handheld computers for health care professionals, but it touches only briefly on the prevalence of handheld use. Therefore, to our knowledge, there has not been any structured review conducted to date that specifically addresses the extent of use of handheld devices and estimated adoption rates. As such, this paper aims to systematically summarize all available survey data on health care providers’ use of PDAs with the view of presenting the best available estimates of current PDA use. This paper also aims to project expected future adoption based on established technology diffusion models. From this information we draw implications for research and practice.

## Methods

For the purposes of this systematic review of surveys, the term PDA is used synonymously to refer to any handheld device. Some examples include the following: Blackberry; Palm operating system devices, which include Palm Tungstens, Handspring Visor, and Sony Clie; and Pocket PC devices, which include the Compaq iPAQ and HP Jordana.

### Data Sources

Surveys were identified as a subset selected from a broader systematic review examining all studies related to handheld devices in health care settings. Thus, initial search strategies and retrieved articles reflected this more extensive focus. This comprehensive literature search was conducted in consultation with an information specialist. The searched bibliographic databases covered both medical and engineering disciplines, including the following eight databases: Medline, Current Contents, Inspec, BA/RRM, Biotechnology, Biological Abstracts, EI Compendex, and EMBASE. The search was restricted to English-language literature published January 1993 (corresponding to the development of the first palm device) to February 2005. An updated search of Medline (PubMed) and EI Compendex (EI Village 2) was run near the project’s completion (January 30, 2006).

Furthermore, the reference lists from included studies were examined in an effort to identify additional surveys not captured in the reference databases. In addition, surveys identified from Google searches and those known to the authors to have been conducted by private market research firms as well as physician groups were nominated for inclusion in our screening.

### Electronic Search Strategy

The intent of searching the biomedical databases was to retrieve *all* studies related to handheld devices in health care. It is for this reason that the word *survey* was not included as a specific term in the original search strategy. The search did include the sample search terms detailed in Appendix 1. The search strategy for engineering databases limited retrievals to those articles relating to both handheld computing and health. All bibliographic databases were searched using subject headings tailored to each database and free-text terms in the titles and abstracts.

### Eligibility Criteria

Surveys were included for this present review if they met the following initial criteria: related to an application in human health care and involved the use of a PDA device; contained original data; written in English (not including abstract or conference proceedings); published after 1993; and specifically reported handheld usage rates (prevalence of PDA use as a metric) in populations of health care professionals who were surveyed about the extent of their PDA use. Although conference proceedings were excluded, if deemed potentially relevant, a cross-check was conducted to see if there was an ensuing journal publication. A survey was not included if the handheld device being evaluated had undergone extensive custom modifications. A final set of unique references was identified and posted to the proprietary Web-based screening system SRS (Systematic Review Software).

### Selection Process

**Figure 1:**
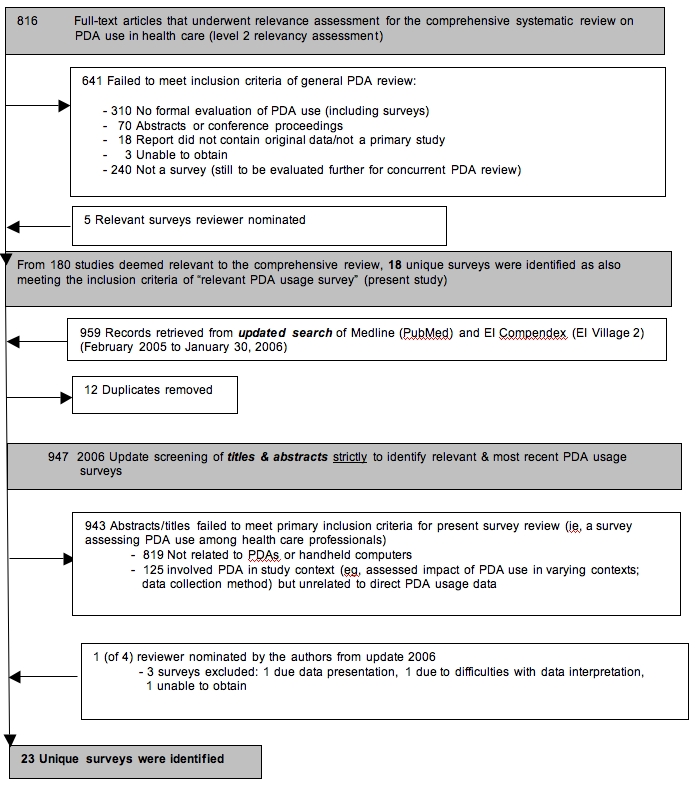
Modified QUOROM Flow Chart for Identified PDA Usage Surveys

The selection process for this present survey review consisted of two phases. First, it began with a screen of full-text articles that had already been retained because their title, abstract, or keywords suggested they contained relevant information on PDA use in health care settings. Therefore, for assessment of relevance, surveys were included if they appeared to contain pertinent study information and if there was no unequivocal reason for exclusion. Second, upon updating the searches, authors returned to the screening of the title, abstract, and keywords for each citation strictly to identify potentially relevant and most recent PDA usage surveys. Eligibility criteria were applied to the full-text surveys, which were reviewed independently by two reviewers (CG and KE). Disagreements were resolved by consensus. Figure 1 provides a modified QUOROM flow chart outlining the process for selecting identified PDA usage surveys.

### Data Abstraction

The contents of each included survey were abstracted by one reviewer (CG), with an additional research assistant providing verification (TR).

### Analysis

The data from all included surveys were extracted in a predefined, standardized fashion with abstraction verified by a second person and assessed descriptively (Appendix 2). Quality assessment methods for descriptive study designs such as surveys have not been established. Although some assessment frameworks exist for assessing survey research [12,13], none of them have been validated or empirically shown to include criteria that are associated with the reduction of bias in empirical surveys. Therefore, survey quality was not formally assessed.

**Table 1:** Included surveys

	**Year of Survey/Publication**	**Author**	**Prevalence of PDA Use**	**Health Care Professional Group**
1	1999/2000	Hucko [18]	15% (use in clinical work)	Physicians
2	NS/2001	ACP-ASIM [19]	47% (use in clinical work)	Specialists (Internists)
3	2001/2001	Versel^*^ [20]	60% (use in practice)	Physician Executives (organizational survey)
4	2001/2001	Martin [21]	19.3% (use in clinical practice)	Physicians & Specialists
5	2001/2001	Taylor [22]	26% (use in practice)	Physicians
6	2001-2002/2002	AAP [23]	38% (NS)	Specialists (Pediatricians)
7	2000-2001/2002	Criswell^*^ [24]	67% (use in practice)	Residents (Family Medicine) (organizational survey)
8	2001/2004	Miller [25]	26.2% (office-based use)	Physicians
9	2001/2004	Balen [26]	33% (use at work or home)	Pharmacists
10	2001-2002/2004	Barrett [27]	75% (use in practice)	Medical Residents
11	2002/2002	Martin [2]	27.9% (use in clinical practice)	Physicians & Specialists
12	2002/2002	Versel^*^ [28]	33% (use in physician offices)	Physician Executives (organizational survey)
13	2002/2003	McCleod [29]	46% (use at medical institutions)	Specialists, Medical Residents, & Fellows (Internists)
14	2002/2004	Carroll [30]	35% (use at work)	Specialists (Pediatricians)
15	2002/2004	DeGroote [31]	61% (use on an academic health science campus)	Health Sciences Faculty & Medical Residents
16	2003/2003	Martin [32]	32.9% (use in clinical practice)	Physicians & Specialists
17	NS/2003	Vincent [33]	36% (use alone or in conjunction with log-card procedure in documenting)	Medical Residents (Family Practice)
18	2003/2003	Versel^*^ [34]	75% (carry & use PDAs)	Physician Excutives (organizational survey)
19	2004/2005	AMA/Forrester [14]	57% (use regularly in a work week)	Physicians, Specialists (Surgeons), & Medical Residents
20	2004/2005	Wilden [35]	91% own; 85% use on daily basis; 9% weekly; 215% monthly	Specialists (Anestheologists)
21	2001/2005	Stromski^*^ [36]	64% of programs report “most or all” residents use for clinical purposes	Medical Resident Programs (Emergency Medicine) (organizational survey)
22	NS/2005	Stroud [37]	67% (NS)	Nurse Practitioners & Students
23	NS/2005	Boonn [38]	45.1% (own or use daily)	Specialists (Radiologists)
	NS/2004	Joy^†^ [17]	Difficult to interpret the prevalence numbers among the resident respondents	Medical Residents (Obstetrics & Gynecology)
	2004/2005	National Physician Survey (Canada)^†^ [15]	Unable to establish overall prevalence due to way data have been presented; 48.6% of medical students have a PDA (although unable to infer use)	Physicians, Specialists (various), & Medical Students
Note: An excerpt from the “Taking the Pulse” study published in October 2004 by Manhattan Research [16] reports that 40% of all US physicians currently use a PDA, increasing from 35% in 2003. However, for this present review, the authors were unable to obtain a full copy of the report in spite of having contacted Manhattan Research on two separate occasions (February 2006).				

NS = not specified

^*^Survey conducted at organizational level (vs individual level responses)

^†^Survey of PDA use but prevalence data could not be established (referred to descriptively only)

## Results

From a total of 816 full-text articles that underwent relevance assessment for a systematic review of the literature examining broad-ranging PDA use in health care, a subset of 18 surveys reporting PDA prevalence rates were identified (see Figure 1). Additionally, upon updating the search, an additional 959 records were retrieved and screened, from which 5 additional unique surveys were included. Furthermore, a total of 8 surveys were reviewer nominated, 3 of which were identified upon updating. Unfortunately, the authors were not able to obtain access to one Internet market research report. Prevalence numbers from 2 surveys were found too difficult to interpret, and, therefore, these data could not be utilized further in our results; however, we refer to both studies descriptively.

It is from this pool of literature that a total of 23 unique surveys were identified (Table 1):15 were published articles in scientific journals, and 8 were nonacademic, reviewer-nominated citations that were either reports available for purchase, press releases, or trade magazine articles and thus not subject to formal peer review. Of these 8 surveys, 5 were conducted by Internet market research firms, 2 were conducted by physician groups, and 1 was conducted by a market research firm in conjunction with a physician group (American Medical Association).

### Survey Characteristics

The included surveys were published between 2000 and 2005, with survey data collected between 1999 and 2004. One survey had a four-year lag between data collection and publication, three surveys had a lag of three years, and three surveys had a lag of two years. We were unable to determine publication lag in four surveys as no data collection dates were provided. Surveys were from the United States (16), Canada (4), Australia (1), both the United States and Puerto Rico (1), and both the United States and Canada (1). Survey methodology reflected the following: self-administered questionnaires distributed solely by mail (11); telephone interviews (2); Web-based online surveys (4); and combined distribution by electronic or postal mail as determined by the recipient (4). Two studies did not report the methodology used. Response rates ranged from 5.7% to 92.6% across 13 of the included surveys; 10 surveys did not report such rates.

### PDA Use

In presenting the results, we group the PDA users by type of health care provider and personal characteristics (eg, age).

In terms of PDA use, physician specialists were surveyed exclusively in five surveys. Three surveys examined practicing physicians, three included physicians and specialists combined, two included medical residents exclusively, while two surveyed an amalgam of physicians, specialists, medical residents, and/or students. Three surveys targeted physician executives and organizational practice leaders. One survey was directed at directors of family practice residency programs, while a further survey targeting individual PDA use in emergency medicine resident programs was completed at the organizational level.

In addition to physicians as users of technology, one survey targeted practicing hospital pharmacists and another targeted a national sample of nurse practitioner students and faculty. One survey included faculty and residents across several health science disciplines, including medicine, dentistry, nursing, public health, pharmacy, and applied health science.

**Figure 2:**
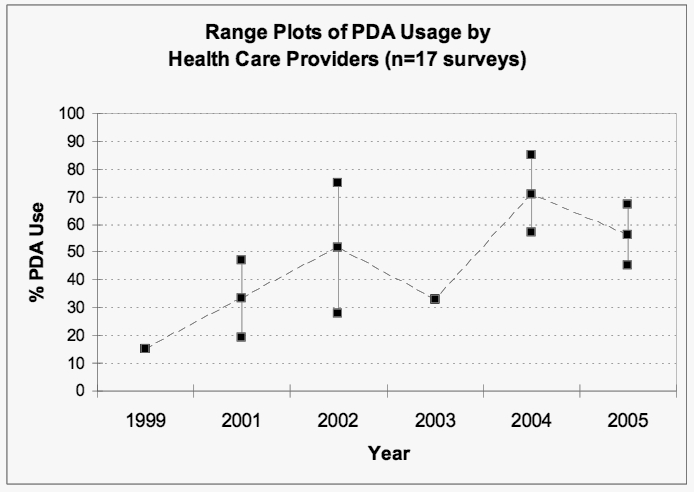
Range plots of PDA usage by health care providers (n = 17); middle points represent range medians

To more accurately reflect handheld use across time, reported surveys were examined, when possible, from the timepoint when survey data were collected versus when published. When not possible, the publication date was the reported timepoint used. Collectively, the included surveys do indicate that PDA use is high, albeit somewhat variable, across studies. The reported prevalence rates of PDA use lend themselves well to an estimation of trend over time (Figure 2), and, as such, since 1999, there is evidence of an increase in PDA usage. Results do not include surveys completed at the organizational level. Surveys are presented according to data collection dates, with the exception of the American College of Physicians study (2001) [19], Stroud (2005) [37], and Boonn (1995) [38], which report publication dates only. The noted drop in 2003 is due to the paucity of surveys conducted in that year. Based on the most recent survey statistics (2004/2005), the current overall adoption rate varies between 45% and 85%, as derived from individual level survey data. In addition, of the five surveys completed at the organization level (eg, physician executives or medical program directors speaking on behalf of their individual members), the PDA use of their group members was estimated to be 60% (2001) [20], 67% (2001) [24], 64% (2001) [36], 33% (2002) [28], and 75% (2003) [34].

To elaborate on the percentage of overall adoptions rates, a US survey of 769 practicing physicians conducted in 1999 found that only 15% of physicians use a PDA in practice [18]. In a 2000/2001 survey of directors of family practice residency programs in the United States and Puerto Rico, use of handheld computers by either an individual or group was reported in 67% of the residency programs [24]. In 2001, 47% of 489 US-based internists surveyed were using a PDA [19]. A subsequent 2001 survey of 834 practicing physicians found that the proportion using PDAs had increased to 26% [22]. If we only look at professional use, then the increase is from 10% in 1999 to 18% in 2001 [22]. Among a national sample of practicing physicians surveyed in 2001, 26% reported using PDAs for office-based work [25]. In 2001/2002, 38% of 696 office-based physicians indicated that they used a PDA in their practice [23]. Of practicing hospital pharmacists surveyed in 2001, 33% reported using a PDA at work or home, with 28% using one on a daily basis [26]. These numbers reflect both types of use: personal and professional (ie, as an integral part of everyday practice). In 2001, 75% of residents in a teaching hospital reported using their PDA on a daily basis [27]. In 2002, 35% of US pediatricians were using a PDA at work, and 40% had one for personal use [30], and 46% of internal medicine physicians and residents were reporting PDA use [29].

In Canada, similar PDA use data have been collected since 2001 as part of the annual Physician Resource Questionnaire conducted by the Canadian Medical Association. PDA use among physicians increased from 19% in 2001 [21] to 28% in 2002 [2] and to a third in 2003 [32]. These data conclude that, in 2003, a third of Canadian physicians were using PDAs, which marked a 73% increase from 2001. Further, more than 50% of Canadian medical doctors under 35 years of age reported that they were using a PDA or wireless device in clinical practice [32]. The data did not differentiate type of professional use.

In a PriceWaterhouseCoopers survey in 2001, 60% of the physician executives who responded indicated that their organization had at least one physician with a PDA [20]. Reportedly, this represented an upward trend from 26% in a similar 2000 survey. Further, in 2003, the trend continued, and 75% of respondents reported that their organization’s physicians were using PDAs. This increase in PDA use came after a steep decline to 33% in 2002 [28,34]. A sample of health science faculty and medical residents was surveyed in 2002 about their PDA use. Combined results from the various faculties and residents indicated that 61% used a PDA [31].

In 2004, 57% of a sample of US physicians indicated that they regularly used a handheld computer in a typical work week [14]. Results obtained in 2004 from a survey of members of the Austalian Society of Anaesthetists indicated that 91% of respondents owned a PDA; 85% reported using it on a daily basis, and 66% were reportedly “dependent” upon the handheld device, although the term *dependent* was not defined [35]. In 2005, when physician members of the Radiologicial Society of North America were surveyed, 45.1% reported owning or using a PDA on a daily basis [38]. However, the survey authors suggested use among this group of specialists appeared to be lower than for other physicians because a radiologist often works in front of a full workstation in clinic and therefore relies less on a mobile device. Further, PDAs are not yet well equipped to handle the tasks radiologists need to perform. In 2005, Stroud et al became the first group of researchers to address the use of PDAs in the field of nursing. Survey results concluded that the majority (67%) of participants used this technology [37].

While PDA use has clearly increased since 1999, it appears as though only a handful of studies have examined the prevalence and usage patterns of such technology outside of physician groups. Furthermore, when comparing the included surveys in depth, distribution of use is not uniform across selected characteristics of surveyed health care professionals. Therefore, further subgroup analyses from the included surveys are provided below. Patterns of handheld use are also briefly examined.

### Patterns of PDA Usage

#### Age

Based on a survey of 250 family physicians, as far back as 1995, younger physicians (less then 40 years of age) were more likely to consider carrying a handheld computer than older physicians (94% vs 84.5%) [39]. More recent data from this present review also suggest an age differential in usage patterns. A 2001 survey of 834 US practicing physicians found that use of handheld devices was higher among doctors under age 45 (33%) than among older doctors (21%) [22]. Another study found that pediatricians graduating from medical school in the last five years were more likely to use a PDA in practice than those who graduated more than five years ago [30]. According to a survey conducted by the American Academy of Pediatricians in 2001, PDA use was highest among those members under 30 years of age, with a reported usage rate of 75% [23]. Another study found that 60% of US internists below 40 years of age used a PDA, while only 34% older than 51 years did [19]. McLeod et al (2003) also found that PDA usage captured in 2002 among a sample of internal medicine physicians and residents under 30 years was much higher (68%) versus those over 40 years of age (37%) [29]. In Canada, 2003 usage was highest among younger physicians, with more than half of those under the age of 35 years (53%) using a PDA, compared with 15% of physicians aged 65 or older [32]. According to the American Medical Association/Forrester Research 2005 Physician and Technology Study, more doctors under the age of 40 years were reportedly using PDAs (55%) than those over 40 years (45%) [14]. In 2005, the mean age of nurse practitioners and students who reported using a PDA was 42 years [37].

#### Students and Medical Residents

Residents tend to be younger, therefore it follows that they are more likely to use PDAs. This is also substantiated by direct evidence. A survey of directors of family practice in the United States and Puerto Rico conducted in November 2000 (306 responses) found that use of handhelds in residency programs, either by an individual or group, was 67% [24]. A 2001 survey of residents in a teaching hospital reported that more than 75% used their PDA on a daily basis [27]. Stromski et al (2005) surveyed emergency medicine residency programs in 2001 to identify the methods of procedure documentation to examine the number of programs transitioning to more advanced information technology systems (eg, PDA use). Their results indicated that 13% of the residency programs required the use of PDAs, 15% of programs purchased PDAs for their residents, and a similar proportion reported that PDAs were used by “most or all” of their residents to document procedures. Further, 64% of programs reported that “most or all” of their residents utilized PDAs for clinical purposes. DeGroote et al found that, in 2002, 71% of medical residents reported using PDAs versus 56% of faculty members [31]. In a 2002 survey, McLeod et al noted that the percent of frequent PDA users among internal medicine residents and fellows in training exceeded 70%, compared to only 50% of attending physicians [29]. From a survey of the experiences of family resident graduates in obtaining hospital privileges and in documenting procedures and deliveries, Vincent et al (2003) concluded that 36% of the respondents used a PDA alone or in conjunction with a log-card, paper-based system. Unfortunately, this study did not present any other prevalence data on PDA use [33]. However, from survey data captured in 2004, the handheld technology gap between residents and physicians began to close: a US study concluded that 73% of residents regularly used a handheld computer in a typical work week, followed closely by 71% of family/general practitioners [14]. In a survey of PDA use by nurse practitioner students and faculty, Stroud et al found that of the total respondents who reported PDA use, 73% were nursing students [37].

One survey by Joy et al (2004) met our initial criteria but could not be incorporated into the results analysis. Although this study did examine PDA use in obstetrics and gynecology residency programs, it was difficult to interpret the prevalence numbers among the resident respondents. Likewise, the National Physician Survey (2004) did not present overall PDA prevalence rates but did ask Canadian medical students if they had a PDA or wireless device [15]. Of the 2721 respondents, 24% in first year, 40.6% in second year, 70.6% in third year, and 71.6% in fourth year reported having a PDA, representing an overall average of 48.6% among students [15]. Unfortunately, these 2004 figures provide no information on how medical students were using this technology and in what contexts.

#### Gender

PDA usage among men and women was equal in a 2001 survey of internists [19]. Similarly, McLeod et al (2002) found no significant gender difference in PDA users among a 2002 sample of internal medicine physicians and residents [29]. However, pediatrician PDA users were most likely male, as reported in 2002 [30]. As well, the 2003 Physician Resource Questionnaire analysis concluded that male physicians were somewhat more likely to use a PDA in their practice than were females (35% vs 30%) [32]. More recent data from a 2004 survey of PDA use among US physicians, specialists, and medical residents suggested that male clinicians were slightly more likely than their female counterparts to regularly use handhelds (53% vs 47%) [14]. On the other hand, nurse practitioner data from 2005 show that men (82%) were notably more likely than women (64%) to use a PDA (*P* < 0.05) [37]. However, the authors cautioned that they were unable to determine the significance of this finding given that the actual survey sample of men (n = 38) as opposed to women (n = 188) was small. The authors suggested that if ease with PDA technology is less common in women, then the nursing profession, dominated by females, may need elevated momentum to adopt PDA technology across nursing practice [37].

#### Family Physicians versus Specialists

The most recent Physician Resource Questionnaire (2003) analysis concluded that Canadian family physicians were just as likely to use a PDA (33%) when compared to medical (34%) and surgical (32%) specialists [32]. This was the third consecutive year these figures rose consistently across all physician groups in Canada [2,21,32]. However, according to a US survey of physicians published in 2005, the biggest adopters of PDAs in professional practice were family and general practitioners (71%) when compared to surgical specialists (54%) [14]. The above mentioned studies are the only survey data available directly comparing general physician use to that of specialists.

#### Large and Hospital-Based Practices

A US survey of practicing physicians found that use was higher among those who were wholly or partly hospital-based (33% and 29%, respectively) than among those who were office-based (23%) [22]. Usage was also higher among physicians in large practices (33%) than in solo practice (16%) [22]. Carroll et al (2004) also found that PDA users tended to not be in private practice [30]. Additional survey data from 2004 indicated that of US physicians practicing in primary practice offices with fewer than 10 physicians, 49% reported regular use of a handheld computer [14]. Miller et al (2004), reporting on a national sample of practicing physicians, found that in a group practice consisting of an average of nine physicians, handheld use was approximately 56% [25].

#### Urban versus Rural Physicians

From a random sample of US pediatricians in 2002, PDA users were most likely from urban communities [30]. Similarly, results from Canada’s Physician Resource Questionnaire in 2001 indicated PDA use to be higher among physicians practicing in urban centers (19.9%) than in rural centres (13.4%) [21]. However, by 2002, rural use (29.6%) surpassed urban use (27.7%) among physicians [2]. In Canada, this trend continued in 2003, with 36.9% of rural respondents indicating PDA use versus 32.5% of urban respondents [32].

#### Professional Use

Five surveys considered PDA use in both a professional and personal context; 17 studies exclusively captured professional use. One study reported general prevalence rates for PDA use among pediatricians; however, it did not specify if use was in clinical practice or outside of work.

In order to discern professional use more closely, we explored administrative PDA uses versus direct use in clinical patient care. We found that of the surveys that concern PDA use within a health care setting, 17 of 23 studies (74%) reported use pertaining to administrative or organizational tasks, while 14 of 23 studies (61%) addressed PDA use in patient care. Billing and coding were the most frequently performed administrative PDA functions in 50% of the surveys reporting administrative uses. This was followed by 44% reporting calendar scheduling, 31% reporting Web and email access, 25% reporting address book use, and 25% stating use in charting patient details into an electronic health record. Other reported administrative tasks included the following: word processing, calculator, charge capture, procedure documentation, outpatient tracking, resident hours, telephone message tracking, general time management/personal organizer, patient referrals, procurement of supplies, patient census, order entry, dictation, and passwords and pins.

In terms of patient care, access to drug information was reported in 93% of the surveys reporting clinical PDA use, while 50% reported prescribing, 43% stated accessing patient records, 43% described medical calculator use, and 36% indicated use in reference to laboratory values. Other reported clinical PDA uses included access to medical references, patient tracking and patient reminders, clinical decision pathways and managed care applications, telemedicine, and diagnostic imaging or radiology applications.

Only one survey reported PDA use for patient education, and one referred to PDA use for research purposes.

## Discussion

This paper summarizes the results from surveys examining adoption of PDA use. These survey data are in reasonably good agreement and suggest a sizable proportion of physicians use handheld devices. However, most of the sources of survey data did not distinguish well between types of applications being used most often and whether the PDAs were being used professionally for administrative purposes or for direct clinical work. It is encouraging to note that our findings are similar to those of an analysis of online registrations and downloads of a PDA drug reference guide, which concluded that approximately one fifth of US physicians (150000) and half of medical students in the United States (33000) were PDA users [40].

Our grouped survey data suggest that there is little information on the PDA usage rates among nonphysician health care providers. However, collectively, these data suggest that use of handheld devices has become a subject that health care professionals need to know about. By systematically gathering this usage information, it is difficult to deny the prevalence of PDAs in health care. With this basic understanding of current handheld usage patterns, we need to consider the impact of this development of mobile handheld technology on both practice and research.

According to a commonly accepted descriptive model of the diffusion of innovations developed by Rogers, when the cumulative rate of users of a new invention is plotted versus time, the result is an S-shaped curve [41]. Interestingly, this appears to be true of most technological innovations, irrespective of the technology. For example, Hall and Khan (2003) reviewed the S-shape adoption patterns of a variety of 20th century consumer products (eg, washing machines, video cassette recorders) [42], while Teng et al (2002) developed historical diffusion curves for information technologies (eg, personal computers, email) [43]. Variations in diffusion slopes do exist given that some technologies will diffuse more rapidly than others.

Health care information technologies have also been examined within this diffusion framework. England et al (2000) studied organizational and technological factors determining the rate at which innovations diffuse in the health industry [44]. In 2005, RAND Health completed a report characterizing the diffusion of electronic health records along an S-shaped adoption curve [45].

**Figure 3:**
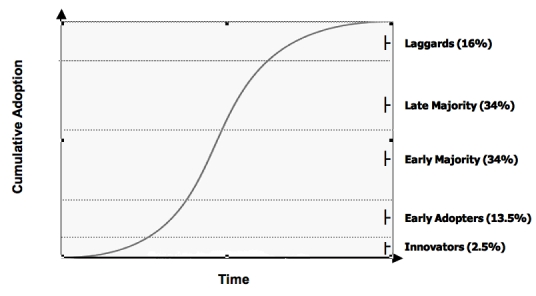
The S-shaped diffusion of technology curve [41]

Technologies typically go through multiple phases during their adoption life cycle, which may last for many years [41,46]. The characteristics of the adopters change over time and so does the nature of suitable evidence to inform their adoption decisions. For example, innovators (the first 2.5% who adopt a new technology) do not need evidence to make an adoption decision. Early adopters (the next 13.5%) are satisfied with case studies and examples of successful adoption and benefits [41]. Examining the typical technology adoption curve for handheld devices (Figure 3) based on the adoption percentage of PDAs thus far from the most recent available data (2004/2005), it can be concluded that we are now at the steepest stage in the adoption S-curve, with a transition from the early majority to the late majority.

The increase in PDA adoption means a potential reduction in hardware and training costs when using handheld devices in the provision of care and in research. Because of the high probability that target health care professionals may already have a handheld device and will already know how to use one, the overall hardware purchase costs could be reduced, and the end user will not necessarily have to be trained from scratch.

To date, use of PDAs in health care appears to have preceded extensive evaluative research. PDA adoption rates, already high, continue to be a moving mark with projections for rapid growth in the short term. By comparing handheld device diffusion to other health information innovations, and by placing PDA use within existing diffusion models, we are able to better predict the future of handheld growth in health care and therefore develop more timely and appropriate evaluative research to accompany such growth.

Unfortunately, we were unable to include information from two national physician surveys. The first report entitled “Taking the Pulse” was published in October 2004 by Manhattan Research [16]. Information gleaned from a report excerpt stated that 40% of all US physicians surveyed in 2004 were using a PDA, marking an increase from 35% in 2003. Reported top activities performed on a PDA by all US physicians (in order) were personal scheduling, professional scheduling, accessing a drug reference database, accessing online information, writing/entering clinical notes, and mobile email access [47]. These report findings are similar to our overall findings in this present review.

The second national physician survey not incorporated into our analysis was the Canadian National Physician Survey (NPS) (2004), which provides valuable insight into what information technology, including PDAs, physicians and specialists have in their main patient care settings [15]. However, overall prevalence rates could not be determined from the data provided given the manner in which they were presented. Nonetheless, in reviewing the national data, we can descriptively draw some conclusions. First, it appears as though male physician PDA use is higher than that of females. This appears to be consistent across all tasks involving PDA use although differences do appear to be small. This is consistent with our general findings in which males are only marginally more likely to use a PDA than are females. Interestingly, when examining age-related data from the NPS, it appears as though the age factor may in fact be PDA task-specific. For example, electronic health record usage appears to decrease as the age of physician users decreases. However, PDA use for drug interaction information increases when the age of the physician user decreases. This appears contrary to most other surveys that show younger age is associated with higher general PDA use. Perhaps what this information tells us is that handheld use may be more complex when broken into task-specific strata.

It is worthy to note that, with the exception of one survey focusing on nurse practitioner students, little mention was made in the surveys of PDA use by students across health care disciplines, including medicine. Several universities in Canada and the United States now mandate use of PDAs for medical undergraduate students and residency programs; therefore, it is assumed this could potentially affect prevalence rates. However, because none of the included surveys examined mandated use, we are unable to infer if this is responsible for recent increases. However, this raises an important issue to be considered in future studies related to students and rates of handheld adoption.

To better understand the prevalence rates among the included surveys, it became important to categorize the drivers for PDA use as either professional or personal. We therefore attempted to discern what specific PDA tasks the respective health care professionals were performing. This was done by classifying, whenever possible, the use as administrative versus care. On the surface, it would appear that administrative and organizational tasks on a PDA exceed those related to patient care, perhaps signaling where the growth in adoption is most likely to occur.

In this present review, we can only speak broadly to rates of adoption and patterns of use. Drawing inferences from the survey data was often limited by lack of, or differences in, operational definitions in aspects of handheld use being measured. For example, the term *use* was often not defined by frequency (eg, specific units of time—day, week, month). Taking these issues into consideration would be a useful exercise for future surveys as well as information technology prevalence studies in health care.

In conclusion, physicians are increasingly accustomed to using a PDA, and, therefore, technology expertise will not likely be a barrier to deploying handheld applications. There is an urgent need to evaluate the effectiveness and efficiency of specific tasks using PDA technology (eg, implementation, searching, reference, data entry, reporting) to inform those persons developing and those using handheld applications. Furthermore, it is not clear why there is a paucity of evidence on the extent of adoption of PDAs by other health care providers: is it that they lag in the use of this technology or is it simply that they have yet to be studied?

### Limitations

This review has a number of limitations. Issues around response bias and inability to draw causal inferences weaken survey methodology. It may be the case that those surveyed feel a stronger affinity to the survey sponsor, who has a greater interest in the questions asked, or are in complete disagreement with the topic at hand. This can skew results in difficult-to-measure ways. Quite possibly, the nonrespondents are the least committed (ie, nonusers of PDAs). As a result, the critical objective of drawing a true random sample of the populations that are the focus of the survey is compromised and the findings somewhat impure.

The reported methodologies across these surveys appear to be heterogeneous, which limits their comparability. As noted, the quality of the included surveys could not be determined given the absence of validated quality assessment instruments, and, therefore, there was no adequate way to assess the influence of bias. A related issue is that some of the included surveys did not go through a rigorous peer-review process. These combined issues made judging the strength of the evidence not possible. One would assume surveys identified from scientific journals would be a source of less biased information. However, in defense of the nonacademic surveys, there is a consistency in results between those peer-reviewed versus those that were not. This may suggest that our main conclusions regarding adoption rates are fairly robust and not disconnected even with the inclusion of non–peer-reviewed evidence.

### Conclusions

The objective of this study was to determine the adoption rates of PDAs in health care settings, and to project expected adoption in the future based on established technology diffusion models. Our findings from a systematic review indicate the current overall adoption rate for professional use of PDAs among health care providers, namely physicians, is 45% to 85%. Younger physicians, residents, and those working in large and hospital-based practices are more likely to use a PDA. Professional use in health care settings appears to be more focused on administrative tasks when compared to those related to patient care, although this requires further study. The adoption rate is now at its highest rate of increase according to a commonly accepted diffusion of innovations model. Additionally, the impact of PDA use on practice appears to be immediate in terms of costs and training. Familiarity will not likely be a barrier to deploying handheld applications in health care. However, there is a critical need to evaluate the effectiveness and efficiency of specific tasks using handheld technology within the health care system and across health care provider PDA user groups.

## Abbreviations

NPS: National Physician Survey
PDA: Personal Digital Assistant

## Appendix 1

### Medline Search Strategy

**Medline Search History (Silver Platter)**

#19 (#17 and (la=english)) or ((#12 and (la=english)) or (#10 and (la=English)))
#18 #17 and (la=english)
#17 (palm or palms) and (microcomputer or computer or software)(157 records)
#16 palm or palms
#15 microcomputer or computer or software
#14 (#12 and (la=english)) or (#10 and (la=English))
#13 #12 and (la=english)
#12 hand held computer
#11 #10 and (la=English)
#10 (handspring or apple newton or jornada) or (windows ce or pocket pc or clie) or (pda or personal digital assistant or personal digital assistants) or (handheld computer) or (palm pilot or palm os) or (blackberry or ipaq)
#9 palm pilot or palm os
#8 (la=english) and #7
#7 (handspring or apple newton or jornada) or (windows ce or pocket pc or clie) or (pda or personal digital assistant or personal digital assistants) or (palm pilot or palm or palms or palm os) or (handheld computer) or (blackberry or ipaq)
#6 blackberry or ipaq
#5 handspring or apple newton or jornada
#4 windows ce or pocket pc or clie
#3 pda or personal digital assistant or personal digital assistants
#2 palm pilot or palm or palms or palm os
#1 handheld computer

							            Additional database search histories are available upon request from the authors.
											

## Appendix 2

**Table:** Characteristics and Results of Surveys of PDA Use by Health Care Providers

**First Author, Publication Year, Country of Origin**	**Year Data Collected**	**Survey Methodology**	**Description of Health Care Professionals**	**Sample Size & Response Rate (RR)**	**PDA Usage Rates**	**PDA Use by Age (%)**	**PDA Use by Gender (%)**	**PDA Use by Setting**
Hucko, 2000, US [18]	1999	Mail survey	Practicing Physicians	769 respondents; RR NR	**15%** use handheld devices in practice	NR	NR	NR
ACP-ASIM, 2001, US [19]	NS	NR	Physicians (Internists)	489 respondents; RR NR	**47%**	< 40 years = 60% 41-50 years = 42% ≥ 51 years = 34%	NR (Stated usage among males & females equal)	NR
Versel, 2001, US [20]	2001	Mail survey	Physician Executives	432 respondents; RR NR	**60%** physicians in their practices	NR	NR	NR
Martin, 2001, Canada [21]	2001	Mail survey	Physicians (General Practitioners/ Family Physicians; Medical Specialists; Surgical Specialists)	For general survey RR = 42%; for PDA question 3246 respondents (992 female/2254 male); RR NR	**Overall use = 19.30%** GP/FP = 15.7% Med spec = 22.9% Surg spec = 22.4%	< 35 years = 26.8% 35-44 years = 20.8% 45-54 years = 19.6% 55-64 years = 17.5% ≥ 65 years = 10.8%	Female = 15.4% Male = 21%	NR
Taylor, 2001, US [22]	2001 (Jan-Feb)	Interviews (type NR)	Practicing Physicians	Nationwide sample 834; RR NR	**26%** (18% main use in practice; 8% mainly personal use)	< 45 years = 33% ≥ 45 years = 21%	NR	Group size: solo practice = 16% 2-9 = 28% 10-24 = 37% ≥ 25 = 33% mostly office-based = 23% mostly hospital-based = 33% exclusively hospital-based = 29%
AAP: Periodic Survey of Fellows #51, 2002, US [23]	2001 (Oct)-2002 (Feb)	Self-administered mail survey	Pediatricians (members of AAP)	1616 surveyed; 54.6% (882)	**38%** of reporting physicians (n = 696) use PDAs Use included: keeping a daily schedule (77%), accessing pharmacology references (76%), and medical calculations (75%)	Use highest among PDA users < 30 years (72%)	NR	100% office-based practice
Criswell, 2002, US & Puerto Rico [24]	2000 (Nov)	Mail survey	Directors of Family Practice Residency Programs	610 directors (493 listed in AAFP; 117 ACOFP) ; 306 respondents (257 AAFP; 49 ACOFP) = RR of 50%	Use of handheld computers either by an individual or group reported **67%** (204/306 programs); 30% of programs require applications used uniformly by all users	NR	NR	NR
Miller, 2004, US [25]	2001 (Oct-Nov)	Interviews (telephone)	Practicing Physicians	National stratified random sample of 1200; RR = 5.7%	**26.2%** used PDAs for work	Specific use by age NR (but mean age 48 years according to Physician IT User Type classification)	Specific use by gender NR (but % male = 81.8% according to Physician IT User Type Classification provided)	Specific use by setting NR (but mean practice size MDs = 8.8; group practice % = 55.8% according to Physician IT User Type Classification)
Balen, 2004, Canada [26]	2001 (May)	Mail survey	Practicing Hospital Pharmacists	106 sampled; 58 completed; RR = 55%	**33%** reported using PDAs at work or home; 28% used device daily	NR	NR	NR
Barrett, 2004, US [27]	2001 (Oct)-2002 (Apr)	Email invitation & online Web-based survey	Medical Residents from 7 residency programs (primary care & specialty programs)	Contacted 223 residents enrolled in six week residency programs; 88 completed survey RR = 40%	**75%** stated daily use of PDA	NR	NR	NR
Martin, 2002, Canada [2]	2002	Mail survey	Physicians (General Practitioners/ Family Physicians; Medical Specialists; Surgical Specialists)	For general survey RR = 37%; PDA question 2882 respondents (912 female/1970 male); RR NR	**Overall use = 27.9%** GP/FP = 25.1% Med spec = 31.1% Surg spec = 30%	< 35 years =43.7% 35-44 years = 31% 45-54 years =28.3% 55-64 years = 22.5% ≥ 65 years = 11.9%	Female = 23.8% Male = 29.7%	NR
Versel, 2002, US [28]	2002	Mail survey	Physician Executives	444 respondents; RR NR	**33%** of physician groups (not individual members)	R	R	NR
McLeod, 2003, US [29]	2002 (May)	Mail survey	Internal Medicine Physicians & Residents	Mailed to 867 (473 returned & completed); RR=55%	Proportion of respondents who reported current PDA use = **46%** (218/473)	< 30 years = 68% 30-39 years = 51% ≥ 40 years = 37%	Female = 38% Male = 48%	Dept. of Internal Medicine at a multi-specialty, tertiary care academic medical center in the US Midwest
Carroll, 2004, US [30]	2002	Mail survey	Pediatricians (including residents)	Random sample of 2130 pediatricians; 1185 responded; RR = 62.3%	**35%** currently use PDA at work; **40%** currently use PDA for personal use	NR	NR Stated users most likely male (AOR = 2.29%, 95% CI 1.64-3.19)	Users most likely in urban community (AOR = 1.81, 95% CI 1.30-2.55) NOT in private practice (AOR = 1.47, 95% CI 1.03-2.11)
De Groote, 2004, US [31]	2002 (Nov)	Email invitation & online Web-based survey	Tenure, tenure-track & faculty and residents (including medical residents; dental, nursing, applied health sciences, public health science, pharmacy, and medical faculty)	1538 sampled; 352 responders; RR = 24%	**61%** used a PDA; 69% stated they owned a PDA	NR	NR	NA
Martin, 2003, Canada [32]	2003	Mail survey or email	Physicians (General Practitioners/ Family Physicians; medical Specialists; Surgical Specialists)	For general survey RR = 28.4%; PDA question 2251 respondents (756 female/1486 male); RR NR	**Overall use = 32.9%** GP/FP = 32.5% Med Spec = 33.8% Surg Spec = 32.2%	< 35 years = 52.6% 35-44 years = 38.7% 45-54 years = 31.1% 55-64 years = 27.8% ≥ 65 years = 14.7%	Female = 29% Male = 34.9%	NR
Vincent, 2003, US [33]	NS	Mail survey	Residents	RR = 62%	**Overall use = NR** Use alone or in conjunction with log-card procedure in documenting = 36%	NR	NR	NR
Versel, 2003, US [34]	2003 (Jul-Aug)	Online Web-based Survey	Physician Executives	436 survey respondents; RR NR	**18%** (78 respondents) indicated **75%** of physicians in their organizations using PDAs; 75**%** report that their organizations have at least 1 physician with PDA	NR	NR	NR
AMA/Forrester, 2005, US [14]	2004 (Aug-Dec)	Mail and online Web-based survey	Physicians (General Practitioners/ Family Physicians; Specialists; Residents/ Students as chosen randomly from AMA’s database)	NR	**57%** used regularly in typical work week (average among all physicians) Use in typical work week: Residents = 73% Family/GPs = 71% Surgeons = 54%	< 40 years = 55% use PDA in typical work week	Female = 47% Male = 53%	Use in typical work week in primary practice (office-based with 10 or fewer physicians) = 49%	
Wilden, 2005, Australia [35]	2004	Email request for Web-based survey	Anesthetists (members of ASA)	1870 sampled; 215 responders; RR = 11% (= 24% of ASA members actively using email)	**85%** use their PDA on a daily basis; 9% weekly; 5% monthly 91**%** own PDA 66% consider themselves “dependent” on PDA	NR (age, gender, and type of practice demographics presented but not in relation to PDA users)				
Stromski, 2005, US [36]	2001	Telephone survey	Emergency Medicine Residency Programs	113/122 programs; RR = 92.6%	**Overall use = NR** 64% of programs report “most or all” residents used a PDA for clinical purposes	NR	NR			NR
Stroud, 2005, US [37]	NS	Questionnaire sent via email or postal mail	Nurse Practitioner Students and Faculty	855 questionnaires distributed; 222 responded; RR = 27%	**Overall use = 67%**	NR (report indicated positive correlation between age and frequency (r = .21, *P* < .05) but stated this explained only 4% of variance)	Females = 64% Males = 82%			NR
Boonn, 2005, US & Canada [38]	NS	Recipients mailed surveys with option to complete by mail or via the Internet	Members of RSNA	1628 surveys sent; RR = 32.4%	**45.1%** reported owning or using a PDA on a daily basis	NR	NR (gender and type of practice demographics presented but not in relation to PDA users)			
PDA = personal digital assistant; NS = not specified; NR = not reported; RR = response rate; NA = not applicable; AAFP = American Academy of Family Physicians; AAP = American Association of Pediatricians; ACOFP = American College of Osteopathic Family Physicians; ACP = American College of Physicians; AMA = American Medical Association; ASIM = American Society of Internal Medicine; ASA = Australian Society of Anaesthetists; RSNA = Radiological Society of North America										
